# A new species of intertidal crab of the genus *Ilyoplax* Stimpson, 1858 (Decapoda, Brachyura, Dotillidae) from the Aghanashini River Estuary, a RAMSAR site on the west coast of India

**DOI:** 10.3897/zookeys.1291.201892

**Published:** 2026-09-01

**Authors:** Mani Prema, Vithal S. Kakati, Peter K. L. Ng

**Affiliations:** 1 Lee Kong Chian Natural History Museum, National University of Singapore, 2 Conservatory Drive, Singapore 117377, Singapore Lee Kong Chian Natural History Museum, National University of Singapore Singapore Singapore; 2 Centre for Marine Living Resources and Ecology, Ministry of Earth Sciences, Kochi, India Centre for Marine Living Resources and Ecology, Ministry of Earth Sciences Kochi India; 3 Centre of Advanced Study in Marine Biology, Annamalai University, Tamil Nadu, India Centre of Advanced Study in Marine Biology, Annamalai University Tamil Nadu India; 4 Central Marine Fisheries Research Institute, Kochi, Kerala, India Central Marine Fisheries Research Institute Kochi India

**Keywords:** Burrowing crabs, description, dotillid crabs, Karnataka, mangroves, mudflat, Ocypodoidea, taxonomy

## Abstract

A new species of dotillid crab, *Ilyoplax kagga***sp. nov**., is described from the Aghanashini River Estuary, a RAMSAR site on the west coast of India. It differs from its closest congener *I. gangetica* (Kemp, 1919) by marked differences in carapace features (shape, anterolateral armature, structure of the stridulatory ridges, and the number and kind of tubercles present), dentition on the carpus and palm of the chela, extent of setae on the ambulatory legs, as well as the structure of the male first gonopods.

## Introduction

The intertidal dotillid crab genus *Ilyoplax* Stimpson, 1858, has 28 recognised species (Ng et al. 2008; Davie and Naruse 2010; Trivedi et al. 2015; Poore and Ahyong 2023), all of which inhabit muddy coastal flats in the Indo-West Pacific region (Kitaura and Wada 2006). Five species are known from the Indian subcontinent, with two from Pakistan (Kemp 1919; Trivedi et al. 2015, 2018).

In 2020, the first author collected specimens of a species of *Ilyoplax* from mudflats in Karnataka on the west coast of India, with more material obtained between 2022 and 2025. Around the same time, the second author also found the same species in the same area. Correspondence between us confirmed that these specimens represented a new species. The present new species of *Ilyoplax* is most similar to *I. gangetica* (Kemp, 1919), which was originally described from Port Canning in the Matla River, Gangetic Delta, West Bengal and later recorded from Maharashtra on the west coast of India (Chhapgar and Borgaonkar 1985) and Phuket Island, Thailand (Kosuge et al. 1994).

In this study, we describe a new species of *Ilyoplax* from the Aghanashini River Estuary in Karnataka. This is significant as this location is a RAMSAR site, which was recently designated for India.

## Material and methods

Fresh specimens were preserved in 70% alcohol and are deposited in the Crustacean Research Laboratory, Centre of Advanced Study in Marine Biology, Annamalai University, Tamil Nadu, India (CASAU). Comparative specimens examined are from the Crustacean Division, Zoological Survey of India, Kolkata, India (ZSIK); and the Zoological Reference Collections of the Lee Kong Chian Natural History Museum, National University of Singapore (ZRC).

Details of all specimens examined are available in the material examined subsection of the systematic accounts below. The morphological characters and terminology mostly follow Davie and Naruse (2010), Garm (2004), and Ng and Ng (2025). The characteristic flat-topped fungiform tubercles on the stridulatory ridge are here referred to as “fungiform tubercles” for convenience. Measurements provided, in millimetres (mm), are of the maximum carapace width (**CW**) and length (**CL**) (taken at the midline from the tip of the frontal margin to the median part of the posterior margin), respectively. Abbreviations used in this study are as follows: **SEM** = scanning electron microscope; **juv**. = juvenile; **G1** = male first gonopod; **G2** = male second gonopod; **P2**–**P5** = pereopods 2–5 (first to fourth ambulatory legs), respectively.

The specimens used for SEM were cleaned gently using a soft brush in 70% alcohol, dehydrated in an ascending series of ethanol and air-dried overnight in a desiccator. The processed specimen mounted on silver stubs using Leit adhesive carbon tabs (12 mm). The micrographs were taken using an environmental scanning electron microscope, ZEISS EVO 15, with an extended-range cascade current detector (C2DX), set at 10 kV.

## Taxonomy

### Superfamily Ocypodoidea Rafinesque, 1815

**Family Dotillidae Stimpson, 1858**

**Subfamily Dotillinae Stimpson, 1858**

#### Genus Ilyoplax

Taxon classificationAnimaliaDecapodaDotillidae

Stimpson, 1858

6D503706-26EC-5D55-B34D-A29A7DC9CBC9

##### Type species.

*Ilyoplax tenella* Stimpson, 1858 (by monotypy). Gender of genus feminine.

##### Remarks.

Davie and Naruse (2010) have discussed the challenge with *Ilyoplax*, noting that the genus as presently defined is clearly polyphyletic, with several distinct lineages. They indicated that a detailed taxonomic revision is required (see also Ng et al. 2008: 235).

#### Ilyoplax kagga sp. nov.

Taxon classificationAnimaliaDecapodaDotillidae

409E777A-8648-5553-9CCF-56B0EFBA13D9

https://zoobank.org/0BD689CB-84A2-4ED7-AB39-0FF9094A0E96

Figs 1, 2A, C, E, G, I, 3, 4A, C, E, G, 5, 6, 7A, C, E, G–I, 8A–E, 9, 10, 11

##### Material examined.

***Holotype***: India • ♂ (4.2 × 2.5 mm); Aghanashini River Estuary, upper mudflat areas in mangroves with soft-muddy substrate and oyster bed, off Karnataka, west coast of India; 14°31'22.46"N, 74°22'51.82"E; M. Prema leg.; 17 Dec. 2020; CASAU CR 1048. ***Paratypes***: India • 9 ♂♂ (5.5 × 3.5 mm, 5.3 × 3.5 mm, 5.2 × 3.3 mm, 5.0 × 3.2 mm, 4.9 × 3.6 mm, 4.8 × 3.3 mm, 4.7 × 3.2 mm, 4.6 × 3.2 mm, 4.6 × 3.1 mm), 11 ♀♀ (5.1 × 3.4 mm, 5.0 × 3.1 mm, 4.8 × 3.1 mm, 4.9 × 3.4 mm, 4.9 × 3.3 mm, 4.7 × 3.1 mm, 4.5 × 3.0 mm, 4.5 × 2.9 mm, 4.5 × 2.9 mm, 4.3 × 2.5 mm, 4.1 × 2.7 mm); Aghanashini River Estuary, upper mudflat areas in mangroves of soft-muddy, and oyster bed habitat, off Karnataka, west coast of India; 14°31'14.31"N, 74°22'35.83"E; M. Prema leg.; 22 Oct. 2025; CASAU CR 1045 • 2 ♂♂ (4.0 × 2.5 mm, 3.6 × 2.6 mm); Aghanashini River Estuary, Kumta, Karnataka; 14°31'22.46"N, 74°22'51.82"E; M. Prema leg.; 12 Dec. 2022; CASAU CR 1046 • 1 ♂ (5.5 × 3.1 mm), 2 ♀♀ (5.0 × 3.5 mm, 4.5 × 2.9 mm); same collection data as previous; CASAU CR 1047a • 1 ♂ (4.5 × 3.0 mm), 2 juv. ♀♀ (4.5 × 3.0 mm, 4.2 × 2.9 mm); same collection data as previous; CASAU CR 1047b • 6 ♂♂ (with subequal chelae, 6.2 × 3.8 mm, 6.0 × 3.9 mm, 5.5 × 3.7 mm, 4.8 × 3.3 mm, 4.6 × 3.2 mm, 4.5 × 3.1 mm); same collection data as previous; CASAU CR 1047c • 10 ♂♂ (6.1 × 4.0 mm, 5.8 × 3.9 mm, 5.8 × 3.7 mm, 5.3 × 3.5 mm, 5.2 × 3.6 mm, 5.2 × 3.4 mm, 5.0 × 3.3 mm, 4.6 × 3.5 mm, 4.6 × 3.3 mm, 4.4 × 3.0 mm), 19 ♂♂ (5.0 × 3.2 mm, 4.7 × 3.9 mm, 4.7 × 2.8 mm, 4.7 × 2.7 mm, 4.6 × 3.3 mm, 4.5 × 3.1 mm, 4.5 × 3.0 mm, 4.5 × 3.0 mm, 4.4 × 3.0 mm, 4.4 × 2.9 mm, 4.4 × 2.9 mm, 4.4 × 2.8 mm, 4.4 × 2.8 mm, 4.4 × 2.7 mm, 4.2 × 3.1 mm, 4.0 × 2.9 mm, 4.0 × 2.7 mm, 3.7 × 2.7 mm, 3.4 × 2.1 mm), 15 ♀♀ (5.4 × 3.4 mm, 5.1 × 3.2 mm, 5.0 × 3.5 mm, 4.9 × 3.3 mm, 4.9 × 3.2 mm, 4.8 × 3.3 mm, 4.8 × 3.0 mm, 4.7 × 3.0 mm, 4.7 × 2.9 mm, 4.6 × 3.1 mm, 4.6 × 3.0 mm, 4.6 × 2.8 mm, 4.4 × 2.5 mm, 4.3 × 3.1 mm, 4.1 × 2.3 mm); Aghanashini River Estuary, upper mudflat areas in mangroves with soft-muddy substrates and oyster beds, off Karnataka, west coast of India; 14°31'09.11"N, 74°22'38.20"E; M. Prema leg.; 22 Oct. 2025; CASAU CR 1047d.

##### Additional material examined.

India • 4 ♂♂ (5.5 × 3.7 mm, 4.9 × 3.0 mm, 4.7 × 3.0 mm, 4.6 × 3.1 mm); Kali River, near Kali Matha Temple, soft-muddy areas in mangroves, Karwar, off Karnataka, west coast of India; 14°50'35.58"N, 74°08'36.22"E; M. Prema leg.; 07 Dec. 2022; CASAU CR 1049. Specimens not suitable to be type material.

##### Comparative material examined.

***Ilyoplax gangetica* (Kemp, 1919). *Holotype***: India • ♂ (5.7 × 3.9 mm); Kaikal, Maree, near junction of Matla and Bidyadhari rivers, Gangetic Delta, West Bengal; S. Kemp leg.; 01 Dec. 1916; ZSIK 9794/10. ***Paratype***: India • 1♀ (5.5 × 3.6 mm); Matla Riverbank, opposite Port Canning, Canning Town, near Kolkata, West Bengal; B. Prashad leg.; 10 Mar. 1918; ZSIK 9808/10. ***Non-type***: Thailand • 1 ♂ (5.6 × 4.5 mm); Ranong, mud, KR(S)’; P. Clark leg.; 11 Nov. 2001; ZRC 2001.2339.

***Ilyoplax stapletoni* (De Man, 1908) *Non-types***: India • 5 ♂♂ (5.2 × 3.7 mm, 5.2 × 3.7 mm, 5.0 × 3.2 mm, 4.2 × 3.3 mm, 4.1 × 3.0 mm), 2 ♀♀ (3.4 × 2.4 mm, 3.2 × 2.4 mm); Diamond Harbour River side, Hooghly River; 22°10'43.0"N, 88°11'37.5"E; M. Prema leg.; 17 Jan. 2024; ZRC 2026.1330. Bangladesh • 3 ♂♂ (6.9 × 4.7 mm, 5.5 × 3.7 mm, 5.3 × 3.6 mm), 3 ♀♀ (6.3 × 4.5 mm, 5.6 × 4.5 mm, 4.8 × 3.6 mm), 1 ovigerous ♀ (5.8 × 4.6 mm); “Jhala Kati, Backergunji dist. Bengal” [Jhalakathi, Jhalakathi District, Barisal Division]; H.E. Stapleton leg.; Jul. 1908; ZSIK 5851/10 ***Syntypes***: • 4 ♂♂ (8.4 × 5.4 mm, 7.9 × 5.3 mm, 6.6 × 4.9 mm, 6.0 × 4.9 mm), 1 ♀ (6.7 × 4.7 mm); Bangladesh and India • Kanaigunji, Backergunji district, banks of Passur River, Khulna, Bangladesh, and banks of Hugli River, near Calcutta (Sibpur, Shalimar, Budge-Budge, and Takta Ghat, West Bengal, India); Jul. 1908; ZSIK 5137/10.

***Ilyoplax frater* (Kemp, 1919) *Syn-types***: Pakistan • 2 ♂♂ (7.6 × 5.2 mm, G1 detached; 5.8 × 3.8 mm), 1 ♀ (4.7 × 3.8 mm); Karachi; collector and date not known; ZSIK 9861/10. ***Non-types***: Kuwait •1 ♂ (5.5 × 3.5 mm); station SDG 57, Kuwait; S. De Grave leg.; 2014/2015; ZRC 2021.0495.

***Ilyoplax stevensi* (Kemp, 1919) *Syn-types***: Pakistan • 1 ♂ (7.6 × 4.8 mm), 2 females (7.3 × 4.9 mm, 4.8 × 3.5 mm), 1 ovigerous ♀ (6.5 × 4.9 mm), 6 ♂♂ (5.7 × 3.7 mm, 4.7 × 3.2 mm, 4.7 × 2.6 mm, 4.5 × 2.6 mm, 3.8 × 2.3 mm, 3.5 × 2.6 mm), 2 ♀♀ (6.0 × 3.6 mm, 4.7 × 2.9 mm); Karachi; collector and date not known; ZSIK 9796/10. ***Non-types***: Kuwait • 2 ovigerous ♀♀ (9.0 × 5.7 mm, 5.6 × 4.5 mm); station SDG 56, Kuwait; S. De Grave leg.; 2014/2015; ZRC 2021.0494.

***Ilyoplax delsmani* De Man, 1926 *Non-types***: Singapore • 1 ♂ (4.5 × 3.7 mm); Sungai Buloh Mangrove, Singapore; P.K.L. Ng leg.; 10 Jan. 1992; ZRC 1992.7927–7935.

##### Diagnosis.

Carapace rectangular, smooth; suborbital margin subovate, lined with evenly spaced small, rounded granules; stridulatory ridge with 22–24 subconical tubercles, inner half of ridge with 5–7 fungiform tubercles, last tubercle being largest, with cap of tubercle fully corneous; epistome triangular. Cheliped massive, carpus short, inner carpal spine blunt, palm broad and lower margin lined with 2 rows of granules gradually increased size towards dactylus; upper margin of dactylus granulated; P2–P4 of similar length, relatively broad, merus length ca. 2.5 times width, tympanum present; carpus and propodus of P2 and P3 with dense patch of plumose setae in male. Female carapace rectangular, suborbital margin subovate, lined with evenly spaced small, conical granules; stridulatory ridge with 22 subconical tubercles, densely arranged, inner half ridge with 4–6 small fungiform tubercles; chelipeds small, subequal; vulvae on sternite 6, comma-shaped, vulvar cover absent.

##### Description.

**Figure 1.**
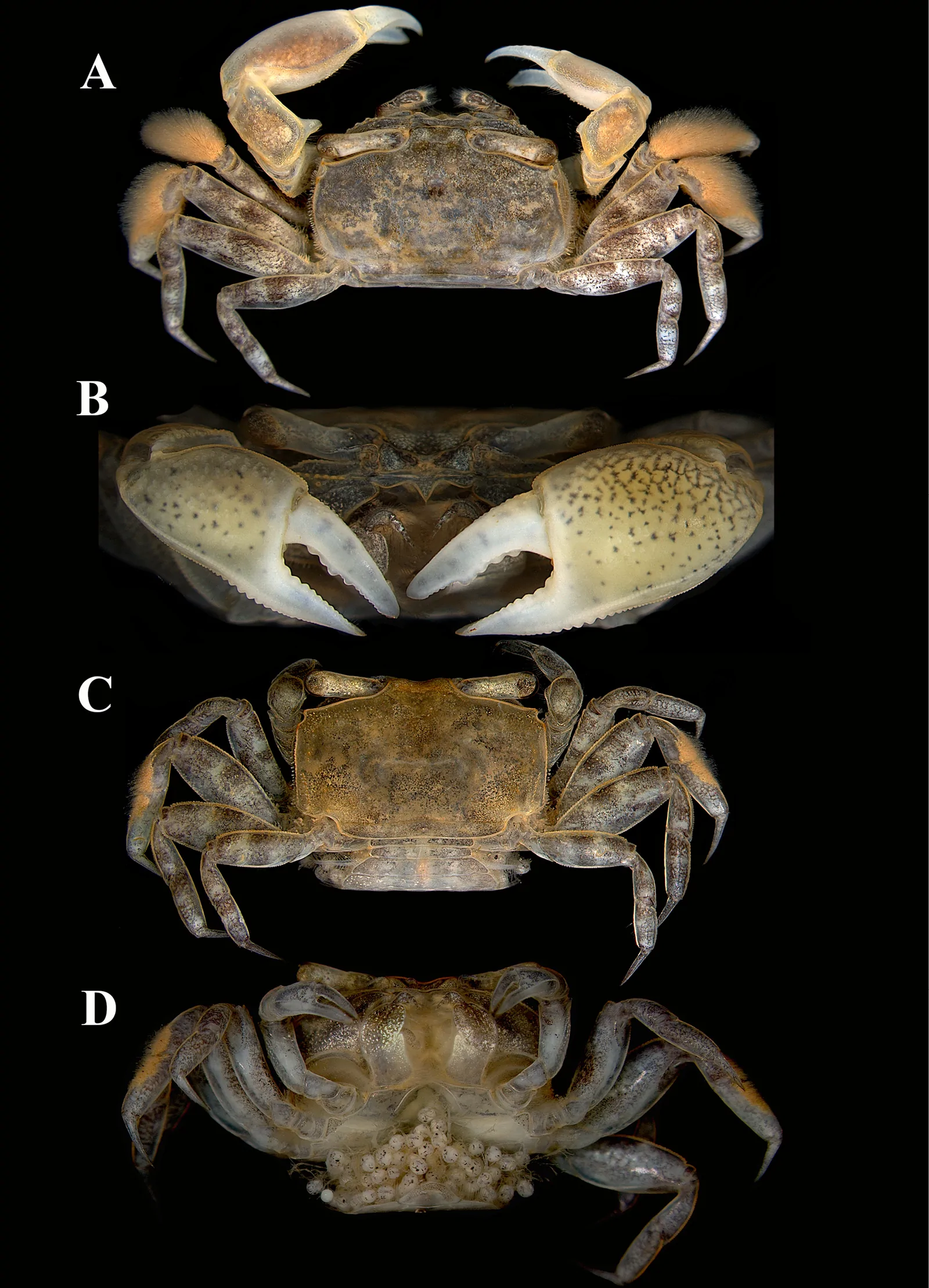
Freshly preserved colouration of *Ilyoplax kagga* sp. nov., Aghanashini River Estuary, west coast of India. **A, B**. Dorsal and frontal views of holotype male (4.2 × 2.5 mm) (CASAU CR 1048), respectively; **C, D**. Dorsal and ventral views of paratype ovigerous female (4.5 × 2.9 mm) (CASAU CR 1047), respectively.

**Figure 2.**
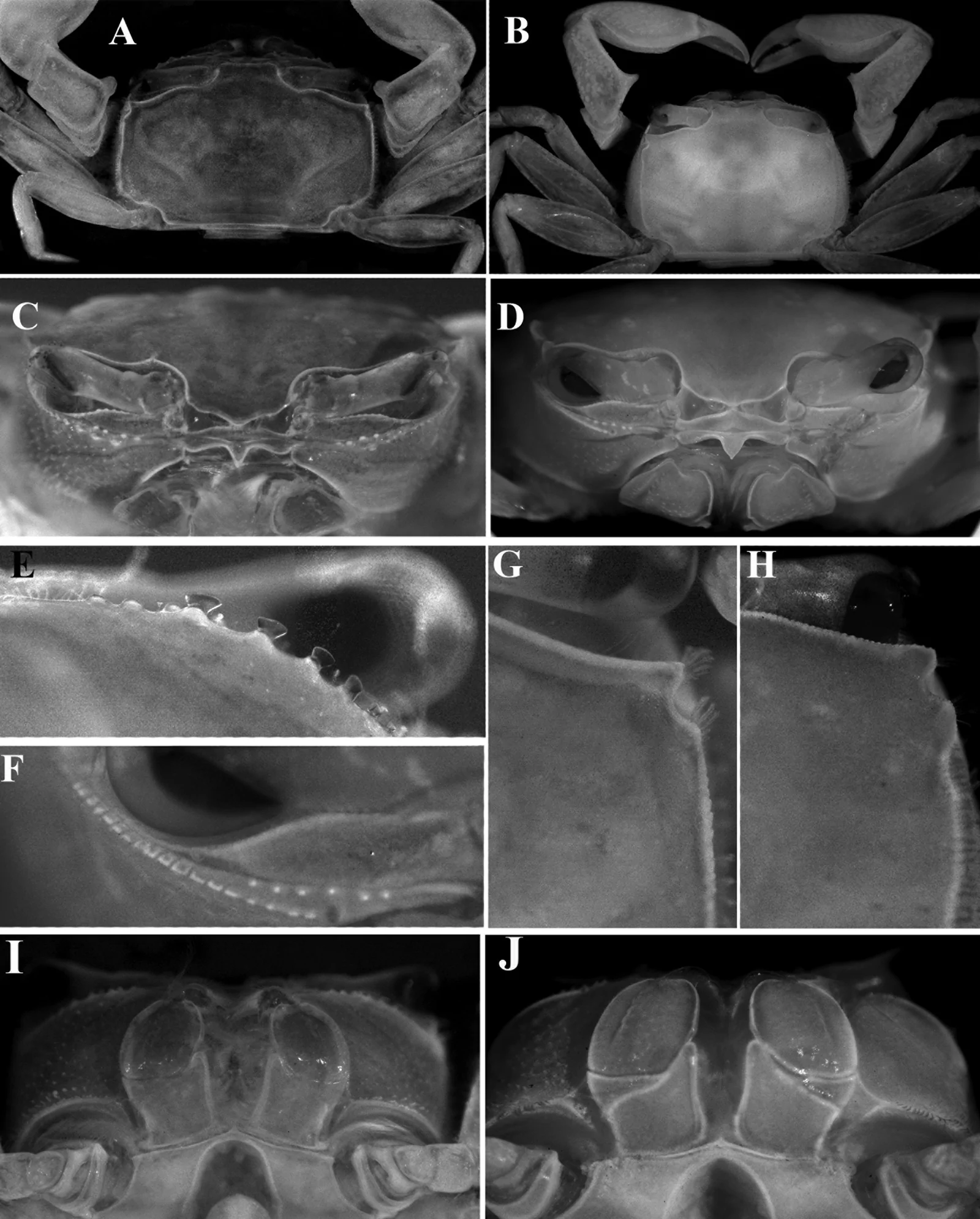
*Ilyoplax kagga* sp. nov. (**A, C, E, G, I**) holotype male (4.2 × 2.5 mm) (CASAU CR 1048), Aghanashini River Estuary, west coast of India. *Ilyoplax gangetica* (Kemp, 1919) (**B, D, F, H, J**) male (5.6 × 4.5 mm) (ZRC 2001.2339), Ranong, Thailand. **A, B**. Dorsal view of carapace and carpus of chelipeds; **C, D**. Frontal view of cephalothorax; **E**. Stridulatory ridge with fungiform corneous tubercles, left side in ventral view; **F**. Stridulatory ridge with pectinated tubercles, right side in ventro-frontal view; **G, H**. Anterolateral teeth of carapace, dorsal view; **I, J**. Maxilliped 3, outer view.

**Figure 3.**
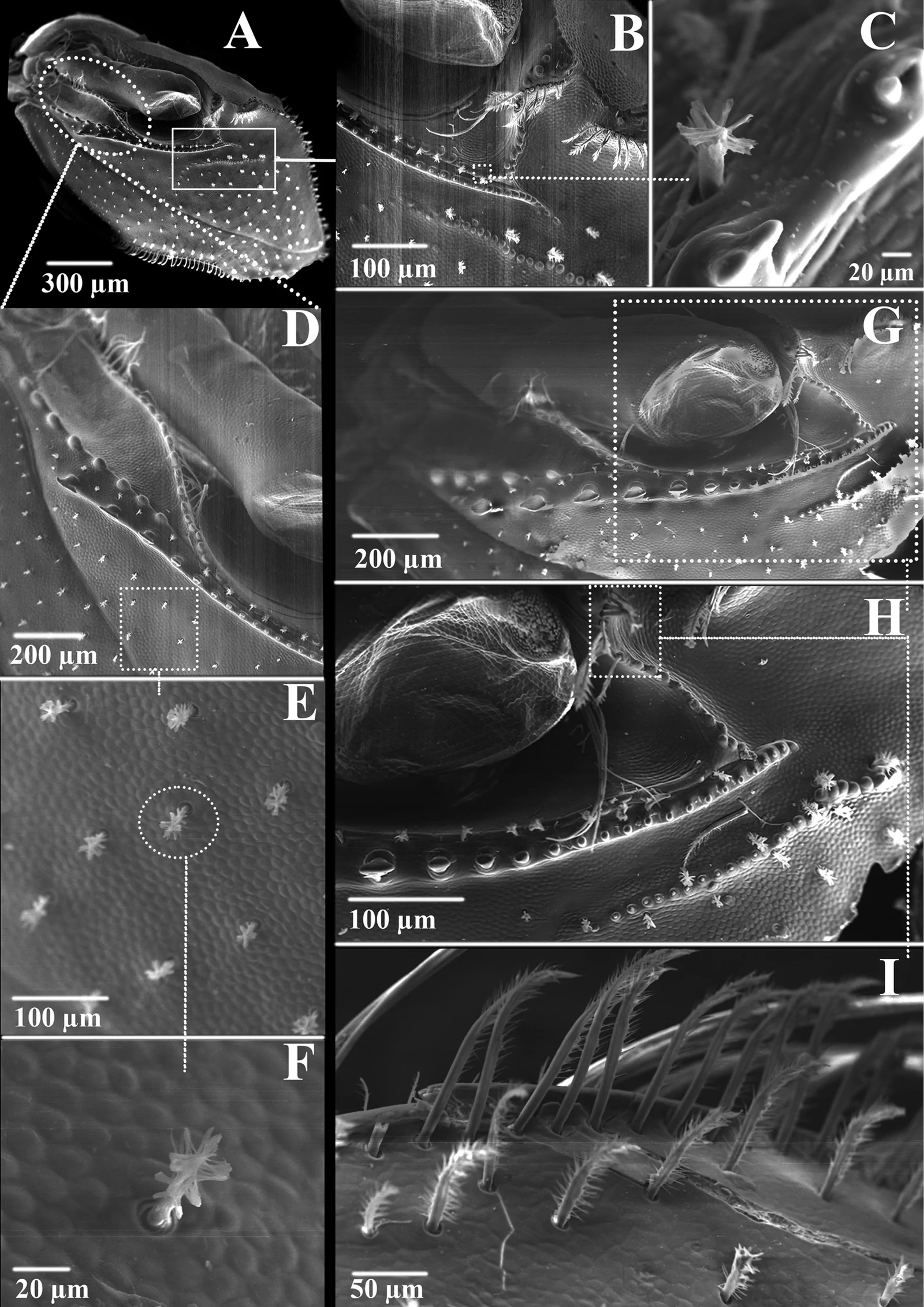
SEM micrographs of *Ilyoplax kagga* sp. nov. (**A–F**) female (5.0 × 3.5 mm), (**G–I**) male (5.5 × 3.1 mm) (CASAU CR 1047), Aghanashini River Estuary, west coast of India. **A**. Pterygostomial region showing evenly spaced setae, suborbital margin and stridulatory ridge; **B, C, G, H**. Stridulatory ridge starting outside lateral edge of external orbit with long papposerrate; **D**. Bifurcated stridulatory ridge and suborbital region, and outer (lower) half of main stridulatory ridge showing 6 fungiform corneous tubercles with 9 conical tubercles; **E, F**. Short stellate setae in different magnifications; **I**. Magnified view of papposerrate setae on external orbital tooth, near to anterolateral teeth.

Carapace (Figs 1A, 1C, 2A, 7A) transversely rectangular, smooth, ca. 1.5–1.6 times wider than long, maximum length medially; regions poorly defined; microscopically granular, with low ridges and transverse setae. Front broad, ca. one-quarter distance between external orbital angles, deflexed, lateral margins converging anteriorly, frontal margin bilobed with gently convex margins when viewed dorsally, median concavity shallow (Fig. 1C). Gastrocardiac groove defined; cardiac region swollen, well defined by clear ridge with anterior margin deeply curved medially, straight, sloping posteriorly. Branchial regions with short transverse ridges or crests, with short stellate setae. Posterior carapace margin straight, broadly rounded laterally, with broad rim; supraorbital margin smooth, sinuous, sloping obliquely; external orbital angle forming acutely triangular, sharp tooth, separated from remainder of margin by distinct concavity; anterolateral margin minutely denticulated (Fig. 2A, G), first anterolateral tooth low, without trace of second tooth or lobe; lateral margins slightly diverging posteriorly. Eyestalks elongated, peduncle thick, short; cornea bulbous (Figs 1, 2C, 3A, C, E).

**Male**: Suborbital margin subovate, lined with evenly spaced small rounded granules; stridulatory ridge immediately below infraorbital margin long, starting outside lateral edge of orbit, appressed against margin medially, before bifurcating around median part of suborbital region (Fig. 3A, B, D, G); outer (lower) half of main stridulatory ridge with 22–24 subconical tubercles, each with corneous tip, gradually becoming larger medially, inner half of ridge with 5–7 fungiform tubercles, last one being largest, with cap of tubercle entirely corneous (Figs 2C, 2E, 3D, 3G, 3H); upper part of bifurcated stridulatory ridge shorter than lower ridge, extending to near base of ocular peduncle, lined with subconical tubercles without corneous tip (Fig. 3D, G); additional short ridge of stridulatory granules below and subparallel to outer part of main stridulatory ridge, with 25 rounded tubercles, each with corneous tip, outer tubercles largest, closely packed, becoming smaller and more spaced out inwards (Fig. 3H). **Female**: Similar to male, except with additional short ridge of stridulatory granules below outer part of main stridulatory ridge with 30 rounded tubercles and curving downwards along inner part (Fig. 3A, B, D).

Suborbital, subhepatic, sub-branchial and pterygostomial regions covered with evenly spaced short stellate setae (Fig. 3A–H); external orbital tooth lined with long papposerrate (Fig. 3I); row of long pappose setae on anterolateral tooth (Figs 2G, 3B, 3G–I).

Maxilliped 3 (Fig. 2I) moderately bulging or convex, completely closing buccal cavity; merus longer than ischium, surface with evenly spaced obliquely stellate setae; antero-internal angle of ischium triangular, apically blunt, rounded, produced along edge of merus; ischium anterior margin deeply curved with line of plumose setae densely arranged, surface with few stellate setae, granules absence.

**Figure 4.**
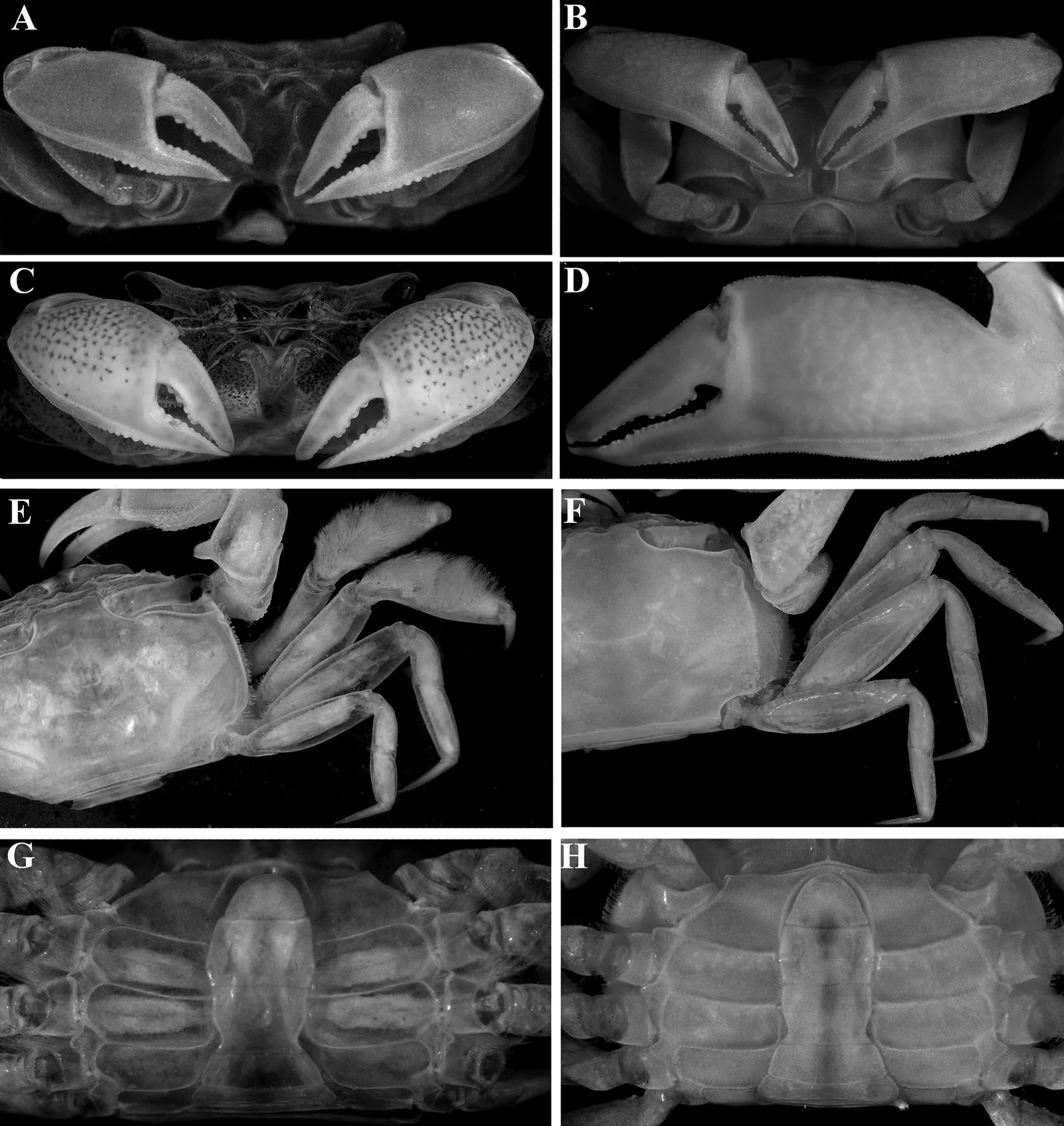
*Ilyoplax kagga* sp. nov. (**A, E, G**) holotype male (4.2 × 2.5 mm) (CASAU CR 1048), (**C**) paratype male with subequal chelae (6.2 × 3.8 mm) (CASAU CR 1047c), Aghanashini River Estuary, west coast of India. *I. gangetica* (Kemp, 1919) (**B, D, F, H**) male (5.6 × 4.5 mm) (ZRC 2001.2339), Ranong, Thailand. **A–D**. Outer view of cheliped; **E, F**. P2–P4, dorsal view; **G, H**. Pleon, telson and sternum.

**Figure 5.**
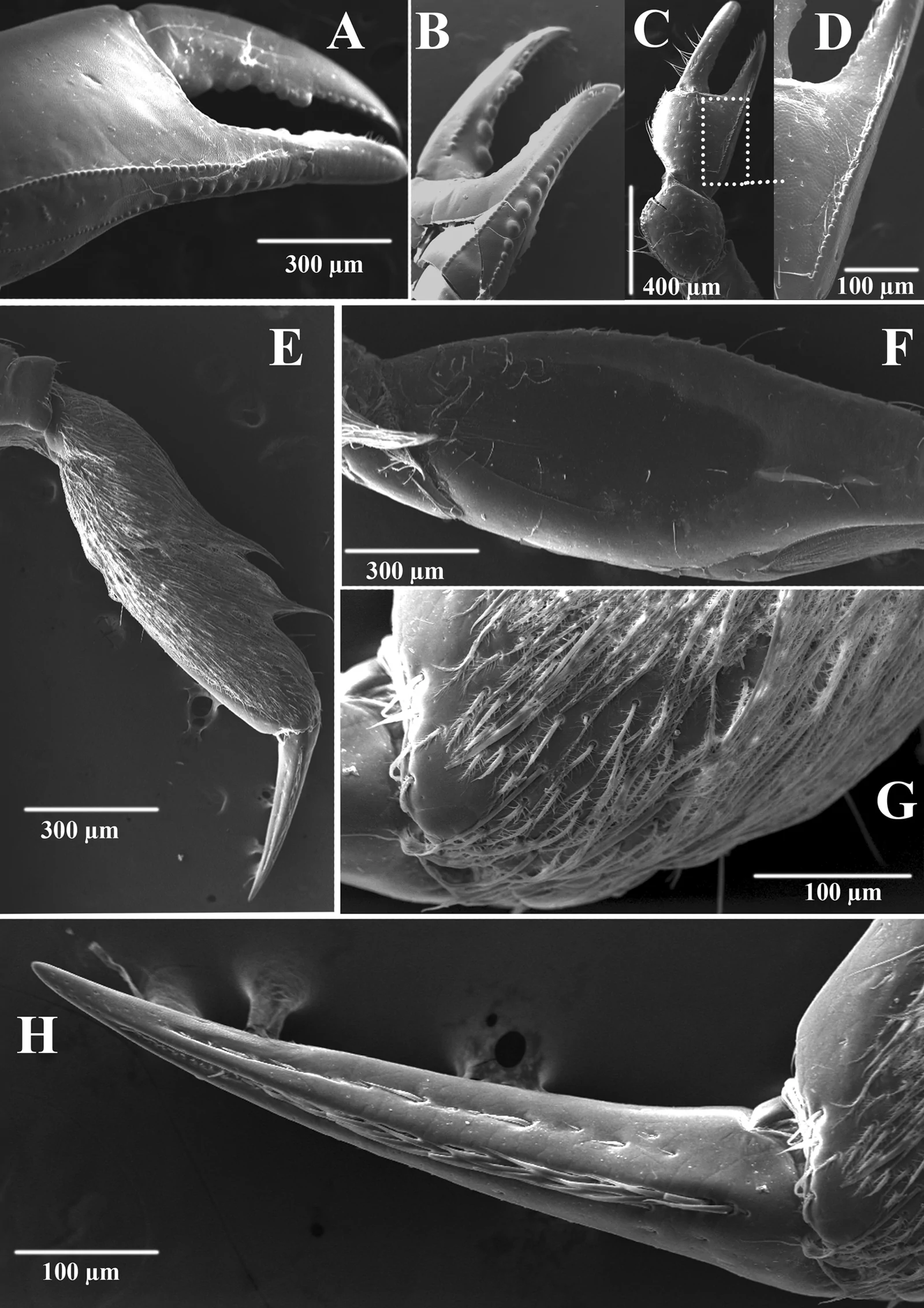
SEM micrographs of *Ilyoplax kagga* sp. nov. (**A**, **B, E–H**) male (5.5 × 3.1 mm), (**C**, **D**) female (5.0 × 3.5 mm) (CASAU CR 1047), Aghanashini River Estuary, west coast of India. **A, B**. Male cheliped, row of rounded, smaller and larger granules on surface of palm; **C, D**. Female cheliped outer view, carpus rounded, granulated margin, and with single row of small rounded granules on palm towards proximal half of fixed finger; **E–H**. Male P3 with tuft of plumose setae, merus upper margin denticulated, tympani present minutely, dactylus with longitudinal grooves.

Chelipeds (Fig. 4A, C) stout, heterochelous; merus trihedral, inner and outer margins granular; carpus short, rhomboidal, armed with short inner tooth. Major chela of adult male holotype distinctly swollen, palm broader, slenderer, margin lined with granules gradually increased size towards dactylus; dactylus upper margin granulated, with broad gap when fingers closed, cutting margin with large, rounded teeth on proximal half and small evenly sized teeth on distal half; pollex cutting margin with small, evenly sized teeth along length (Fig. 5A, B); minor chela similar in form to major chela but proportionately more slender (Figs 1B, 4A). Female chelipeds (Figs 5C, 5D, 7C) small, subequal; carpus rounded, inner carpal spine short; palm with 1 row of rounded granules, tip of dactylus and pollex spatulate.

**Figure 6.**
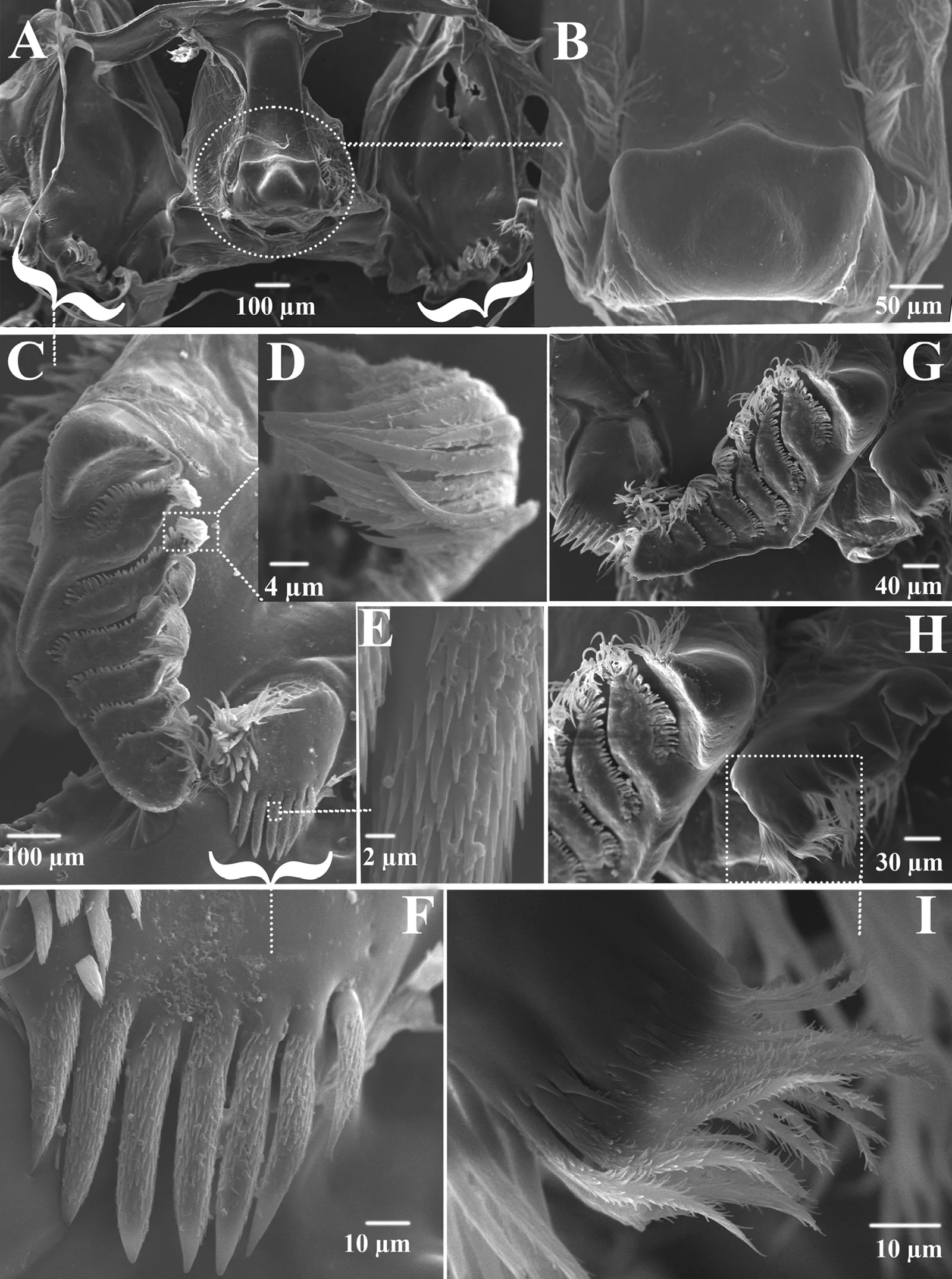
SEM of gastric teeth and microstructures of the associated setae of *Ilyoplax kagga* sp. nov., male (5.5 × 3.1 mm) (CASAU CR 1047), Aghanashini River Estuary, west coast of India. **A**. Overall view of gastric mill; **B**. Smooth median tooth with 3 spines on each side; **C, D**. Lateral tooth anterior margin with row of short bristle-like setae, and lateral edge with tuft of serrulate setae, right side, dorsal view; **E, F**. 7 branched palm-like structure with scaled macrosetae present on posterior margin of lateral tooth; **G–I**. Lateral tooth anterior margin with row of short bristle-like setae, and lateral edge with tuft of serrulate setae, and 7 branched palm-like structure in posterior margin and cusp-like structure on anterior margin; **H, I**. Magnified view of cusp-like structure and long, curved, tuft of serrate setae.

**Figure 7.**
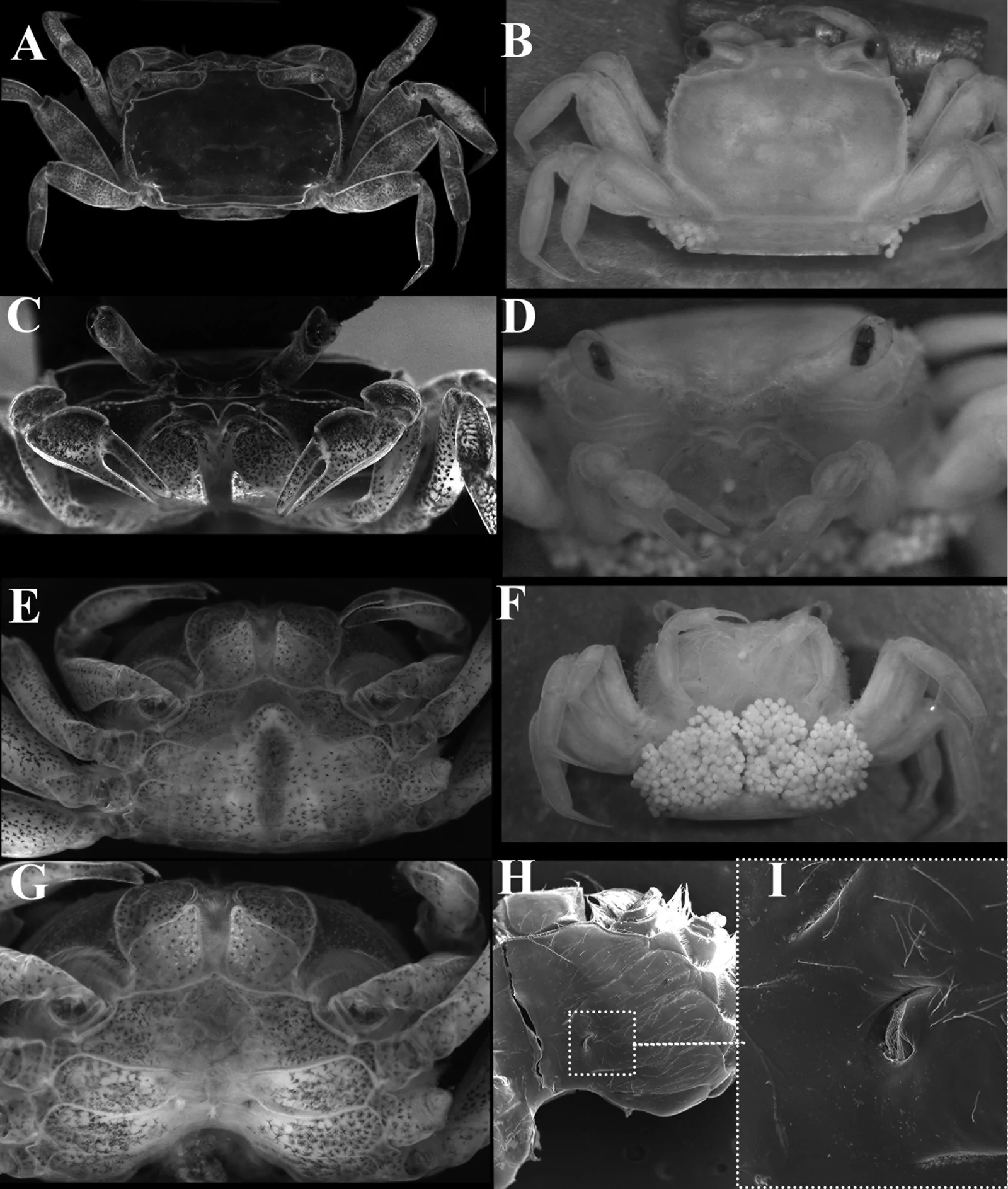
**A, C, E, G, H, I**. *Ilyoplax kagga* sp. nov., paratype, female (5.4 × 3.4 mm) (CASAU CR 1047d), Aghanashini River Estuary, west coast of India; **B, D, F**. *Ilyoplax gangetica* Kemp, (1919), paratype, ovigerous female (5.5 × 3.6 mm) (ZSI 9808/10), Matla Riverbank, opposite Port Canning, West Bengal, India; **A, B**. Dorsal view; **C, D**. Frontal view of cephalothorax; **E–G**. Sterno-pleonal view; **H, I**. SEM micrograph of sternum and vulvae.

P2–P4 length subequal, relatively broad, P5 shortest. P2–P4 merus length ca. 2.5 times width, about as long as carpus and propodus combined, P2–P5 merus margins denticulated inner surface tympanum present (Fig. 5F); dactylus slender, smooth, longitudinal grooves with row of simple setae, tapering to point, shorter than propodus (Figs 5E, 6E–H, 7A); carpus and propodus of P2 and P3 with dense patch of setae in male (Figs 4A, 5E, 5G), female with less dense bed of setae only on carpus and part of propodus of P3, absent on P2 (Figs 1C, 1D, 7A).

Gastric mill: median tooth plate (Fig. 6A, B) broad, smooth surface, pentagonal, surface slightly convex laterally, convex medially, simple structure, consisting of 1 tooth, each side of lateral margin with 3 curved, pointed, simple spines. Lateral tooth plate (Fig. 6C–I) consisting of 8 teeth, including larger tooth with cup-like structure; each tooth with row of short, bristle-like setae anteriorly and tuft of long, serrulate setae on lateral edge (Fig. 6C, D); lateral tooth palm-like with 7 closely appressed branches, each branch densely covered with scaled macrosetae (Fig. 6C, E, F); anteriorly with cusp-like fang structure comprised of triangular plate, each with long, curved, tuft of serrate setae on edges (Fig. 6G–I).

Male thoracic sternum wide; male sternopleonal cavity reaching to base of maxilliped 3 (Fig. 2I); male telson (Fig. 4G) elongate, apically rounded, slightly shorter than pleonal somite 6; pleonal somite 5 much shorter than pleonal somite 6, markedly constricted at base; pleonal somites 3 and 4 similar, wider at base forming triangular margin laterally; pleonal somites 1 and 2 similar in width and subparallel to posterior margin of carapace (Fig. 4G). Female pleon relatively narrow, not fully covering sternum, with short, semicircular telson; female sternum wide, sparsely distributed plumose setae (Fig. 7E, G, H); female sternopleonal cavity broad, smooth, vulvae located medially on sternite 6, divergent towards median region, simple, comma-shaped, vulvar cover absent (Fig. 7I).

**Figure 8.**
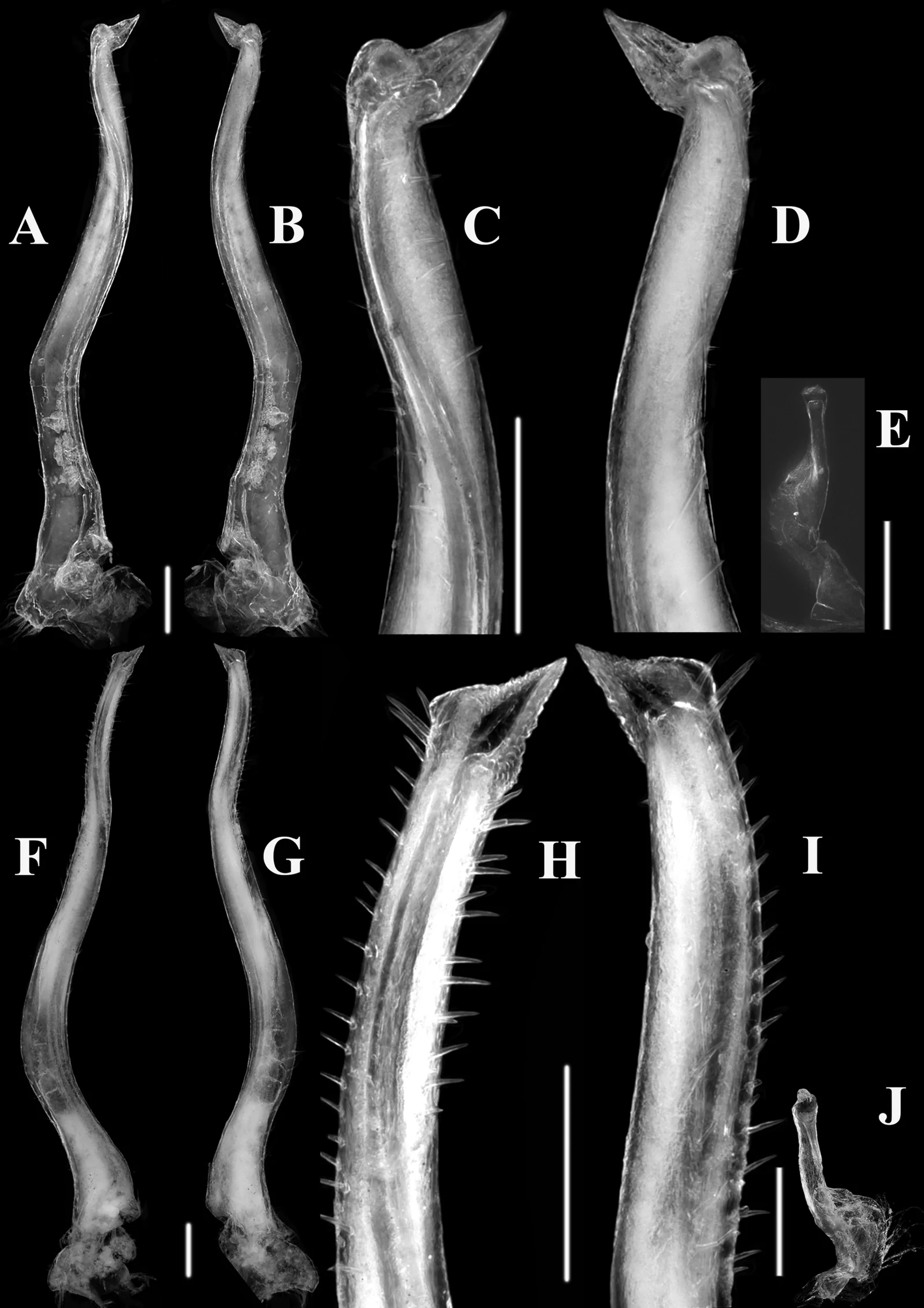
**A–E**. *Ilyoplax kagga* sp. nov., holotype male (4.2 × 2.5 mm) (CASAU CR 1048); **F–J**. *Ilyoplax gangetica* (Kemp, 1919), male (5.6 × 4.5 mm) (ZRC 2001.2339), Ranong, Thailan; **A, F**. Left G1, dorsal view; **B, G**. Left G1, ventral view; **C, H**. Left G1, distal tip, dorsal view; **D, I**. Left G1, distal tip, ventral view; **E**. Right G2, dorsal view; **J**. Left G2, dorsal view. Scale bars: 0.2 mm (**A–D, F–I**); 0.1 mm (**E, J**).

**Figure 9.**
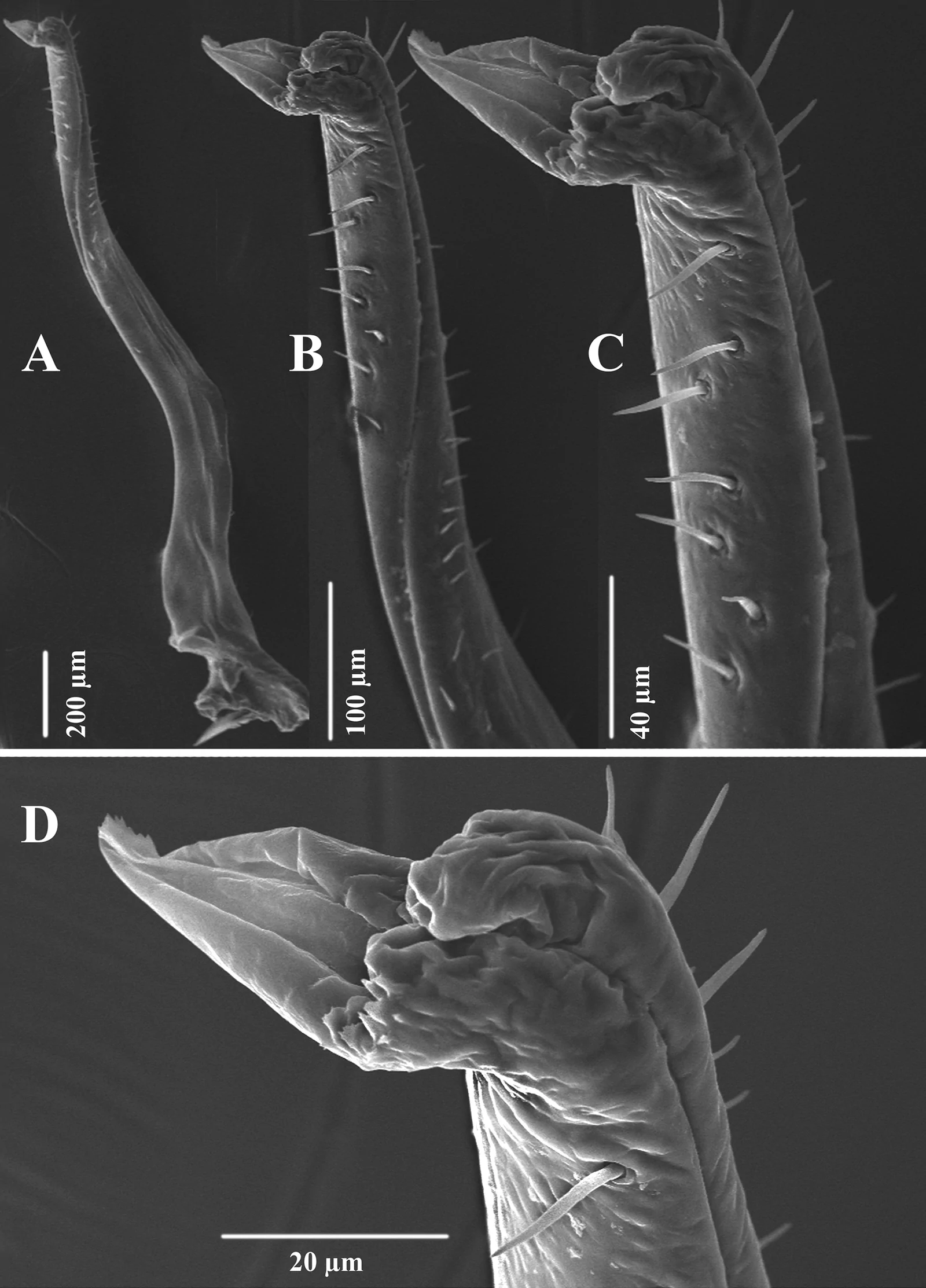
SEM of G1 of *Ilyoplax kagga* sp. nov., paratype, male (5.5 × 3.1 mm) (CASAU CR 1047), Aghanashini River Estuary, west coast India. All images are from left G1, same position at different levels of higher magnification. **A**. Dorsal view of G1; **B, C**. Distal region of G1 with stiff simple setae; **D**. Swollen subdistal region with 2 lobes formed by a median groove.

G1 long, slender, sinuous, proximal third gently bent at angle; distal part prominently elongate, triangular, gently curved, bent obliquely outwards at ca. 90° from longitudinal axis of G1 distal portion; subdistal part prominently swollen just before bend, rounded (Figs 8A–D, 9A–D). G2 short with broad base and slender distal part (Fig. 8E).

##### Variations.

Carapace in larger males have the ridges lined with more stellate setae and with tufts of short setae clearly visible in the frontal view, with surface more densely packed with minute granules; supra orbital margin microscopically granulated in young males more prominent in larger specimens; chelipeds subequal in few males of both large and small size; palm broader, surface smooth, equal in size, with the lower margin lined with granules that are gradually increased size towards dactylus, and the upper margins of palm and dactylus denticulated; some males have the margin of the dactylus and adjacent part granulated. In both males and females, the number of tubercles on the stridulatory ridge after the bifurcating inner (upper) part varies between 5–8.

##### Etymology.

*Ilyoplax kagga* sp. nov. is named in recognition of an endemic rice “Kagga”, a highly salt-tolerant variety that is cultivated traditionally and only in the type locality, Aghanashini village. The name also honours the many generations of ethnic farmers who have preserved and cultivated this endemic rice variety through traditional practices. The specific epithet is used as a noun in apposition.

##### Live colour.

**Figure 10.**
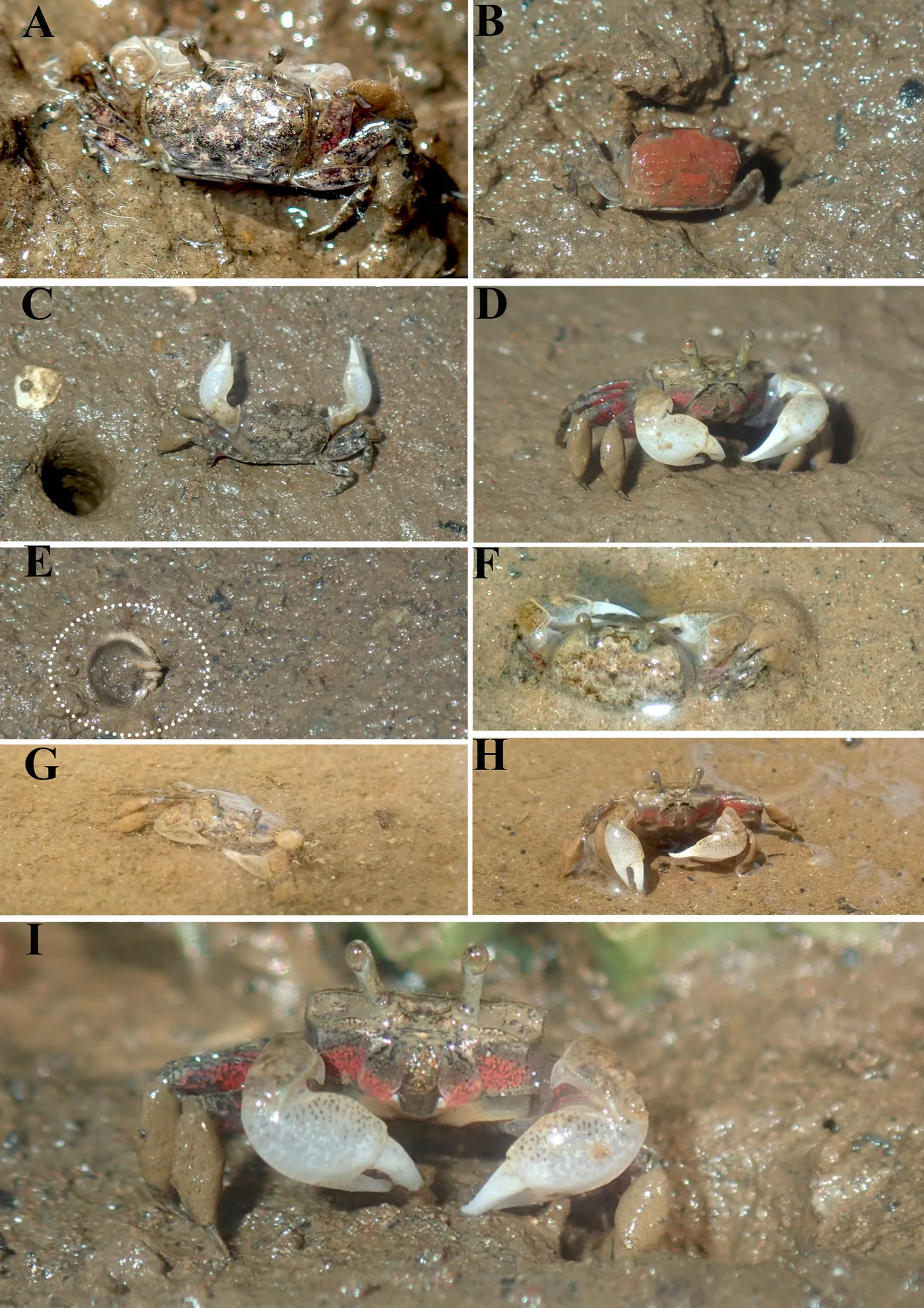
Live colour of *Ilyoplax kagga* sp. nov. **A**. Male (4.6 × 3.1 mm) (CASAU CR 1049), Karwar, near Kali Matha Temple, from Island mangroves with *Rhizophora*; **B**. Female with brownish carapace; **C**. Waving behaviours of male individual; **D**. Crab feeding near to its burrow with tuft of setae on first two ambulatory legs clearly visible; **E, F**. Burying in sandy-muddy habitat; **G, H**. Submerged in water and feeding; **I**. Pterygostomial region, inner side of ambulatory legs pigmented with mottled red pattern; **B–I**. Specimens were not collected.

Male dark brown dorsally; reddish on inner side of ambulatory legs, P2 and P3 with tuft of soft, plumose setae densely arranged on outer surface of carpus and propodus is pale-whitish in colour (Fig. 10A); outer surface of the chela pale brownish-green palm when freshly frozen (Fig. 1B) and more white with brown spotted pattern in life (Fig. 10). Female carapace greenish brown when freshly frozen (Fig. 1C) and reddish in life (Fig. 10B); chelipeds pale white with blackish network pattern (Fig. 1C, D).

##### Habitat.

**Figure 11.**
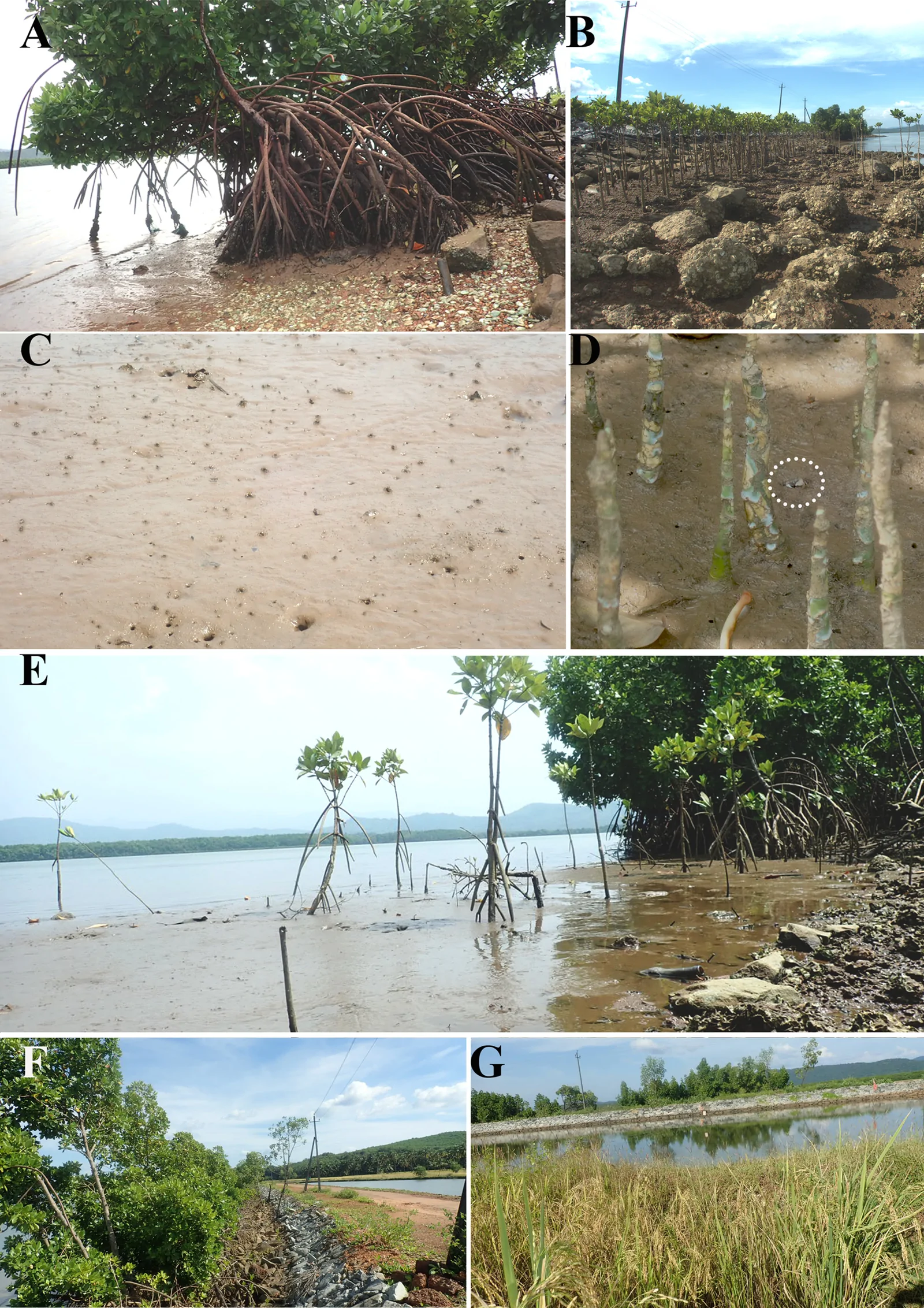
Habitat of *Ilyoplax kagga* sp. nov. at Aghanashini River Estuary, a RAMSAR site. **A**. Upper mudflat bordered by *Rhizophora* mangroves. **B**. Newly planted *Rhizophora* along upper mudflat area; **C**. Soft-sediment mudflat area at low tide; **D**. Crab (white dotted circle) feeding on surface among pneumatophores of *Rhizophora*; **E**. Mudflat area at low tide with young *Rhizophora*; **F, G**. River bordered by mangroves and paddy field of endemic Kagga rice variety adjacent to the mangroves.

The new species was collected from the upper tidal regions of the mangroves and oyster beds. It lives in the burrows of muddy-sandy bottom in the upper limit of intertidal areas. This species is often difficult to collect because of its small body size, cryptic colouration and burrowing behaviour (Figs 10E–G, 11C, 11D), often remaining concealed in narrow burrows and emerging only briefly under suitable tidal and environmental conditions for feeding (Fig. 10G, H). We found this species in large numbers only in Aghanashini River Estuary (Figs 10, 11), with much fewer individuals in the Islands of Kali River Estuary at Karwar.

##### Distribution.

*Ilyoplax kagga* sp. nov. is currently known from the coastal villages of Aghanashini near Kumta and Kali River Estuary near Karwar on the west coast of India, Karnataka, Arabian Sea. The species might also occur in the Mandavi River Estuary, Goa, on the west coast of India.

##### Remarks.

The genus *Ilyoplax* Stimpson, 1858, contains 28 recognised species, of which five species are known from the Indian subcontinent (Ng et al. 2008; Davie and Naruse 2010; Trivedi et al. 2015, 2018; Poore and Ahyong 2023). In general appearance, *Ilyoplax kagga* sp. nov. is most similar to *I. gangetica* (Kemp, 1919) but can be easily distinguished by the following characters: (1) carapace rectangular, smooth, ca. 1.5–1.6 times wider than long, with the upper orbital margin smooth and the lateral parts minutely granulated (Figs 1A, 1C, 2A, 7A) (vs. carapace subrectangular, ca. 1.2–1.4 times as wide as long, with the upper orbital as well as lateral margins microscopically beaded/minutely granulated in *I. gangetica*; Figs 2B, 4F, 7B); (2) external orbital angle more acute and sharp, followed by a relatively broader and deeper concavity, with the first anterolateral tooth low and no second tooth visible (Figs 2G, 3E) (vs. external orbital angle more obtuse, wider, followed by a narrow and deep concavity, with the first tooth low but broad and separated from very low second tooth by shallow concavity in *I. gangetica*; Figs 2H, 7B); (3) epistome slightly more elongated longitudinally, with the median lobe relatively narrower and more triangular (Figs 2C, 4A, 4C) (vs. epistome longitudinally shorter and the median lobe proportionately broader and truncate in *I. gangetica*; Figs 2D, 7D); (4) stridulatory ridge starting outside lateral edge of orbit, bifurcated around median part of suborbital region, outer (lower) half of main stridulatory ridge with 22–24 subconical, corneous tubercles, inner half of ridge with 5–7 fungiform tubercles, last tubercle being largest, with cap of tubercle entirely corneous (Figs 2C, 2E, 3D, 3G, 3H), upper part of bifurcated stridulatory ridge lined with subconical tubercles without corneous tip, additional short ridge of stridulatory granules below and subparallel to outer part of main stridulatory ridge, with 25 rounded tubercles in male (Fig. 3G, H), being similar for the female except that the additional ridge has 30 tubercles which is longer and curves downwards along the inner part (Fig. 3A, B, D) (vs. stridulatory ridge starting outside lateral edge of orbit, not bifurcated, with a single row of stridulatory ridge of 22–25 pectinated comb-like teeth, densely arranged, gradually becoming larger inwards and is more spaced out, without any trace of an additional short ridge in both males and females of *I. gangetica*; Fig. 2F); (5) adult male chelipeds usually heterochelous (Fig. 4C), the carpus being relatively broader and shorter with the inner distal spine more rounded, the palm being short, broad, with the lower margin lined with 2 rows of rounded granules, the lower margin of the fixed finger and the upper margin of the movable finger gently serrated (Figs 1A, 1B, 2A, 4A, 4C, 5A–D) (vs. adult male chelipeds subequal, carpus distinctly elongate and more slender, inner distal spine sharp, the palm being relatively longer, narrower, the lower margin with 2 rows of rounded, minute granules all the way to fixed finger, upper and lower margins of dactylus and propodal fingers minutely serrated in *I. gangetica*; Fig. 4B, D; see also Kemp 1919: 341, fig. 20; Chhapgar and Borgaonkar 1985: 227, fig. 1); (6) ambulatory legs proportionately longer, merus slender with tufts of setae present on the carpus and propodus of P2 and P3 in male (Figs 1A, 4E, 5E, 5G), the setae being absent on P2 in females and present but less dense on P3 (Figs 1C, 7A) (vs. ambulatory legs proportionally shorter, merus broad with the tuft of setae absent or minutely present in *I. gangetica*; Figs 4F, 7B; see also Chhapgar and Borgaonkar 1985: 227, fig. 1); and (7) G1 long, slender, sinuous, with the proximal third appearing gently bent at an angle, distal part prominently elongate, triangular, gently curved, bent obliquely outwards at angle of ca. 90° from longitudinal axis of G1 distal portion, subdistal part just before bend prominently swollen, rounded (Fig. 8A–D) (vs. G1 very long, more slender, more strongly sinuous, distal part relatively short, triangular with straight margins, gently directed obliquely outwards at angle of ca. 70° from longitudinal axis of G1 distal portion, subdistal part just before bend relatively low, obtusely triangular, rounded but not swollen in *I. gangetica*; Fig. 8F–I; see also Chhapgar et al. 2004: 185, fig. 1).

*Ilyoplax kagga* sp. nov. shares some characteristics with other congeners. For instance, the cheliped, epistome and stridulatory ridge pattern of *I. kagga* sp. nov. resemble those of *I. delsmani* De Man, 1926, and *I. stapletoni* (De Man, 1908). *Ilyoplax delsmani*, however, has a very long spine on the inner angle of the carpus of the cheliped (see De Man 1926: fig. 10), but this is completely absent in *I. stapletoni* (present study; see De Man 1908: pl. 18, fig. 1); in *I. kagga* sp. nov., the spine is short and rounded (Figs 1A, 2A). The corneous tubercles on the stridulatory ridges, with their sizes gradually becoming larger medially, are similar, but the shape of the tubercles is significantly different. The tubercles are conical with a pointed tip in *I. delsmani* (see De Man 1926: fig. 3), conical and rounded in *I. stapletoni* (present study), and fungiform in *I. kagga* sp. nov. (Figs 2E, 3A, 3B, 3D, 3G, 3H).

## Supplementary Material

XML Treatment for Ilyoplax

XML Treatment for Ilyoplax kagga

## Additional information

### Artificial Intelligence (AI) use

The authors accept full responsibility for the content of the manuscript, including the disclosure of any use of AI.

No AI tools were used in the preparation of this manuscript.

### Author ORCIDs

M. Prema https://orcid.org/0000-0003-2694-3034

V.S. Kakati https://orcid.org/0009-0005-1336-8167

P.K.L. Ng https://orcid.org/0000-0001-5946-0608

### Data availability

All of the data that support the findings of this study are available in the main text.

## References

[B1] Chhapgar BF, Borgaonkar SS (1985) Extension of range of the estuarine crab *Ilyoplax gangetica* (Kemp) to the west coast of India. Journal of the Bombay Natural History Society 82(1): 226–228.

[B2] Chhapgar BF, Desai BG, Patel SJ (2004) On two interesting marine crabs (Decapoda: Brachyura) from Mandvi, Kutch. Journal of the Bombay Natural History Society 101(1): 184–185.

[B3] Davie PJF, Naruse T (2010) A new species of *Ilyoplax* (Decapoda, Brachyura, Dotillidae) from Panglao, the Philippines. In: Ng P, Castro P, Davie P, Richer de Forges B (Eds) Studies on Brachyura: A Homage to Danièle Guinot. Crustaceana Monographs 11. Brill, Leiden, 75–82. 10.1163/ej.9789004170865.i-366.54

[B4] De Man JG (1908) The fauna of brackish ponds at Port Canning, Lower Bengal. Part X.—Decapod Crustacea, with an account of a small collection from brackish water near Calcutta and in the Dacca District, Eastern Bengal. Records of the Indian Museum 2(3): 211–231 [pls 18–19]. 10.26515/rzsi/v2/i3/1908/163332

[B5] De Man JG (1926) *Ilyoplax delsmani* n. sp., a new species of Ocypodidae. Zoologische Mededelingen, Leyden 9(1): 16–27.

[B6] Garm A (2004) Mechanical functions of setae from the mouth apparatus of seven species of decapod crustaceans. Journal of Morphology 260(1): 85–100. 10.1002/jmor.10213 15052599

[B7] Kemp S (1919) Notes on CrustaceaDecapoda in the Indian Museum. 12. Scopimerinae. Record of the Indian Museum 16: 305–348. 10.5962/bhl.part.25926

[B8] Kitaura J, Wada K (2006) New species of *Ilyoplax* (Brachyura: Ocypodidae: Dotillinae) from the Philippines and Indonesia: behavioral, molecular and morphological evidence. Raffles Bulletin of Zoology 54(2): 373–379.

[B9] Kosuge T, Murai M, Poovachiranon S (1994) Breeding cycle and mating behaviour of the tropical ocypodid *Ilyoplax gangetica* (Kemp 1919) (Crustacea, Brachyura). Tropical Zoology 7(1): 25–34. 10.1080/03946975.1994.10539238

[B10] Ng PKL, Ng PYC (2025) *Colimene*, a new genus for three species previously assigned to *Dynomene* Desmarest, 1823 (Crustacea, Decapoda, Brachyura, Dynomenidae). Zootaxa 5737(3): 349–371. 10.11646/zootaxa.5737.3.3 42407875

[B11] Ng PKL, Guinot D, Davie PJF (2008) Systema Brachyurorum: Part I. An annotated checklist of extant brachyuran crabs of the world. Raffles Bulletin of Zoology [Supplement] 17: 1–286.

[B12] Poore GCB, Ahyong ST (2023) Marine decapod Crustacea: a guide to the families and genera of the world. CSIRO Publishing, Clayton South, xii + 916 pp. 10.1071/9781486311798

[B13] Trivedi JN, Soni GM, Trivedi DJ, Vachhrajani KD (2015) A new species of *Ilyoplax* (Decapoda, Brachyura, Dotillidae) from Gujarat, India. Journal of Asia Pacific Biodiversity 8: 173–177. 10.1016/j.japb.2015.02.005

[B14] Trivedi JN, Trivedi DJ, Vachhrajani KD, Ng PKL (2018) An annotated checklist of marine brachyuran crabs (Crustacea: Decapoda: Brachyura) of India. Zootaxa 4502(1): 1–83. 10.11646/zootaxa.4502.1.1 30486044

